# Sports Industry 5.0: reimagining sport through technology, humanity, and sustainability

**DOI:** 10.3389/fspor.2025.1640362

**Published:** 2025-12-04

**Authors:** Dag Øivind Madsen, Ekaterina Glebova

**Affiliations:** 1 Department of Business, Marketing and Law, USN School of Business, University of South-Eastern Norway, Hønefoss, Norway; 2 Faculty of Business, Higher Colleges of Technology, Dubai, United Arab Emirates; 3 Université Paris-Saclay CIAMS, Orsay, France

**Keywords:** Sports Industry 5.0, industry 4.0, digital transformation, sustainability, social justice, fan engagement, inclusion, ethics

## Abstract

The sports industry faces converging pressures from digitalization, climate change, and social demands for inclusion and well-being. Existing “Sports 4.0” approaches emphasize efficiency and performance but mostly overlook broader social goals. This paper introduces *Sports Industry 5.0* as a conceptual framework that adapts the principles of Industry 5.0 to sport. Unlike prior accounts, we argue that Sports Industry 5.0 is not only an extension of technological innovation but also a reorientation toward human-centricity, sustainability, and resilience across all levels of sport, from elite to grassroots and eSports. The article synthesizes interdisciplinary literature and global examples, while critically addressing risks such as surveillance, greenwashing, and inequities of access. Tables illustrate how Industry 4.0 and 5.0 principles diverge in sports, and case illustrations show how these ideas can be operationalized. We conclude by outlining future research directions, including comparative case studies and cross-cultural analyses, to assess whether Sports Industry 5.0 can function as a universally relevant paradigm.

## 1 Introduction

The sports industry is a globally significant sector, encompassing professional leagues, community recreation, infrastructure development, broadcasting, and retail, and is deeply woven into cultural life. In recent years it has faced unprecedented pressures. Global crises like the COVID-19 pandemic, climate change, economic shocks and social justice movements have exposed vulnerabilities in sports' traditional models. At the same time, disruptive technologies (artificial intelligence, Internet of Things, blockchain, virtual reality, etc.) are transforming how athletes train, how games are officiated, and how fans engage. Observers note that the sports industry is “undergoing a profound transformation, driven by the rapid evolution of digital technologies,” (p. 1) from AI and blockchain to the metaverse, all of which are “redefining how sport is played, consumed, and commercialized” (p. 1) (1). Yet this digital upheaval raises critical questions: can sports leverage innovation while also advancing ecological sustainability, social equity and the wellbeing of all stakeholders?

To address these challenges, we introduce Sports Industry 5.0 as a guiding concept. Industry 5.0, as defined by the European Commission, is a vision of industry “beyond efficiency and productivity,” emphasizing human-centricity, sustainability, and resilience (2, 3). In parallel, *sports*—as a human and social institution—must transcend a narrow focus on performance or profits, embracing broader societal goals (fairness, inclusion, environmental responsibility) alongside technological prowess. This paper integrates diverse literature (management science, sports sociology, technology policy, environmental studies, etc.) to sketch Sports Industry 5.0. We argue that the sports sector can harness digital innovation and meet evolving ethical and social demands. By comparing Industry 4.0 vs. 5.0 principles (4, 5), examining new sports technologies and digital media, and critically addressing fan culture, inclusion, mental health and sustainability, we outline a blueprint for sports' future. Examples are drawn worldwide, from elite clubs and mega-events to grassroots programs and media platforms, to illustrate how a human- and planet-centered approach can reshape sports organizations and practices.

The scope of Sports Industry 5.0 is deliberately broad. It encompasses not only elite professional leagues but also grassroots and community sport systems, recognizing that technological and social transformations affect all levels of practice. eSports and digital gaming are also included, as they now function as both cultural and commercial extensions of sport, with their own governance and inclusion challenges. By clarifying this scope, we aim to avoid ambiguity about the boundaries of the concept.

The novelty of this contribution lies in reinterpreting Industry 5.0 through the lens of sport. While debates on “Sport 4.0” already examine digital transformation, they remain largely focused on technological adoption. By contrast, Sports 5.0 integrates technological change with normative commitments—human well-being, inclusion, and ecological responsibility—positioning sport as both a beneficiary and a driver of wider societal transformation. This theoretical articulation moves beyond description toward a critical framework that can guide future research and practice.

The rest of the article proceeds as follows. Section 2 presents the methodological note. Section 3 compares Industry 4.0 and Industry 5.0 in the sports context. Section 4 examines technological innovation as the foundation of Sport 4.0. Section 5 addresses ethics, data, and human agency, while Section 6 turns to fan culture and digital engagement. Section 7 discusses inclusion, diversity, and social justice, and Section 8 focuses on athlete mental health and well-being. Section 9 examines sustainability and environmental responsibility, and Section 10 considers strategic innovation and governance. Section 11 offers critical reflections on the limits, tensions, and cautions of Sports 5.0. Section 12 concludes the paper.

## 2 Methodological note

This article is conceptual in nature. It synthesizes insights from interdisciplinary literatures in management, sports studies, technology policy, and sustainability. Sources were identified through searches in Scopus, Web of Science, and Google Scholar using combinations of terms such as “Industry 5.0,” “Sport 4.0,” “digital transformation in sport,” “sustainability in sport,” and “sports governance.” The review emphasized recent contributions in English-language scholarship, complemented by selected earlier works where historically relevant. Reports from international institutions (e.g., European Commission) and practitioner analyses (e.g., Deloitte) were included where they contribute to understanding emerging practices. The conceptual synthesis was guided by a comparative lens: examining how principles associated with Industry 4.0 and Industry 5.0 manifest in sport, and identifying both opportunities and tensions. We acknowledge the limitation that non-English literature and grassroots practices from underrepresented regions are less extensively covered, and suggest that future empirical research should address these gaps.

## 3 Industry 4.0 vs. industry 5.0 in the sports context

We first clarify the conceptual shift. Industry 4.0 (4IR), a concept launched in Germany in 2011 (6), refers to the current wave of digital industrial technologies: cyber-physical systems, Internet of Things (IoT), big data analytics, robotics and AI, leading to highly automated factories and service systems (7, 8). Analogously, “Sports Industry 4.0” can be seen wherever sport has embraced these tools—for example, using IoT wearables and data analytics in training, automated systems in sports manufacturing, and even robotic referees or ball-detecting sensors. As Devecioğlu (9) notes, sports institutions have been significantly influenced by developments associated with the Fourth Industrial Revolution, with many sports products and services now closely linked to emerging technologies. High-performance athletes routinely use sensor-laden equipment, teams rely on machine-learning analysis for strategy, and broadcasters employ real-time data feeds.

However, Industry 4.0's emphasis on efficiency and automation comes with trade-offs. Manufacturing-focused Industry 5.0 literature argues for a rebalancing toward human needs and social purpose (10, 11). The European Commission's “Industry of the Future” initiative highlights that future industry should not only optimize production but also address societal goals—climate change, resource efficiency, worker skill development and job satisfaction (2, 3).

While the European Commission has been central in shaping Industry 5.0 discourse (4), similar visions are emerging elsewhere. In Japan, Industry 5.0 debates are linked to the “Society 5.0” agenda, which frames technology as a tool for solving demographic and social challenges (12, 13). In the Americas, discussions around terms such as “human-centric digital transformation” have gained traction in sports management research and among professional leagues experimenting with athlete data governance. In Africa and parts of Asia, grassroots sports projects highlight resilience and inclusion, often using mobile technology to extend access in low-resource settings. Incorporating these perspectives ensures that Sports 5.0 is framed as a global paradigm rather than solely a European import.

In sports, this suggests a transition from purely tech-driven performance to a model that places athletes, coaches, staff and communities at the center, ensuring their well-being and social value. In other words, Sports Industry 5.0 means using advanced tech *with* an unwavering focus on human-centric design and sustainability.

Table 1 contrasts key dimensions of Industry 4.0 vs. 5.0 principles applied to sports. Under the 4.0 paradigm, sports organizations prioritize efficiency, productivity and cutting-edge performance analytics. Athletes and workers must adapt to highly automated processes (for example, accepting data-driven coaching or automated decision systems) while the environmental impact of events is often a secondary concern. In contrast, a Sports Industry 5.0 approach explicitly balances innovation with people and planet: the primary focus is on human wellbeing, equity and ecosystem health, using technology as an assistive tool rather than a replacement of human agency. Sustainable practices (green venues, waste reduction) and resilience to disruptions (like having flexible digital engagement models when crowds are banned) become core, not peripheral.

**Table 1:** Comparison of industry 4.0 and industry 5.0 principles in a sports context (adapted from general industry literature).

Dimension	Industry 4.0 (sports)	Industry 5.0 (sports)	Illustrative cases/stakeholders/outcomes
Primary focus	Efficiency, automation, performance optimization	Human wellbeing, sustainability, and broader social goals	4.0: Automated match analysis systems in elite football to optimize performance.
			5.0: IOC integrating athlete well-being programs alongside competitive excellence.
Technology use	Automated systems, AI analytics, IoT sensors	Human–AI collaboration, assistive robotics, immersive tech (AR/VR)	4.0: VAR technology automating referee decisions.
			5.0: AI-assisted coaching in NBA, where machine insights support rather than replace coaches.
Role of humans	Workers adapt to technology (specialized roles)	Empowered athletes/coaches with upskilled, creative roles	4.0: Analysts providing machine-generated reports to coaching staff.
			5.0: Athlete co-design of wearable tech to improve usability and comfort.
Sustainability	Secondary (focus on resource efficiency)	Central (circular economy models, green operations)	4.0: Energy-efficient LED stadium lighting.
			5.0: Tokyo 2020 Olympics commitment to recycled medals and net-zero venues.
Resilience	Vulnerable to shocks (rigid planning)	Built-in flexibility (adaptive digital engagement, backup systems)	4.0: COVID-19 cancellations exposing fragility of live-only models.
			5.0: Hybrid tournaments with eSports and virtual attendance ensuring continuity.
Examples	Automated training platforms, data-driven strategies	AI-assisted coaching, eco-friendly smart stadia, community fitness apps	Stakeholders: clubs, fans, sponsors, NGOs. Outcomes: cost savings (4.0), inclusion and sustainability gains (5.0).

The table illustrates a fundamental shift: Sports 4.0 emphasizes machine-centric efficiency, whereas Sports 5.0 integrates technology with empathy and sustainability. For instance, where a 4.0 approach might implement a fully automated physical therapy robot, a 5.0 mindset ensures that robot training augments a therapist's guidance and also considers the patient's comfort and consent. Similarly, data collected from fans or athletes in Sports 5.0 is used not just to boost profits but also to improve experiences in ethical ways (e.g., personalized support that respects privacy).

At the same time, applying Industry 5.0 principles to sport should not obscure power imbalances. Access to advanced technologies is highly uneven: elite leagues and wealthy nations can invest in smart stadiums and AI coaching, while many community clubs lack basic infrastructure. Without redistribution, Sports 5.0 could reinforce a two-tier system where innovation benefits a privileged few. Moreover, the rhetoric of human-centricity risks being co-opted as a branding exercise rather than a genuine shift in values. These tensions must be acknowledged from the outset, as they shape how Industry 5.0 principles can realistically be applied in sport.

In the next sections, we explore how these general principles translate into specific technological, cultural and policy developments in the sports world.

## 4 Technological innovation in sports (industry 4.0 foundations)

Sports are already embracing Industry 4.0 technologies on many fronts (14, 15). Modern training programs, for example, rely on IoT and wearables to continuously monitor biometric data (16, 17). An athlete might wear smart garments and wristbands that stream heart rate, movement and fatigue metrics to coaches in real time, enabling immediate feedback. These IoT devices, coupled with big-data analytics, allow coaches to tailor training loads and prevent injury (16, 18). For example, the NBA has adopted wearable biometric monitors to track player fatigue and recovery, raising debates about who owns the data and how it can be used in contract negotiations. In European football, the Video Assistant Referee (VAR) system illustrates both the power and limitations of automation: while improving decision accuracy, it has also sparked controversy about disrupting the flow of the game and disempowering referees (e.g., 19, 20). Blockchain-based fan tokens issued by clubs such as Paris Saint-Germain or FC Barcelona demonstrate how digital tools can open new revenue streams, but also highlight risks of speculation and unequal fan access (21, 22). These cases show that technological adoption is not only about efficiency but also about balancing competing values and interests.

Likewise, computer vision and AI have entered sports (23, 24): camera systems track player positions and ball trajectories to generate advanced statistics (even automated foul or offside detection), and machine-learning models analyze opponents' patterns for strategy. In manufacturing and logistics, robotics and additive manufacturing (3D printing) are used to produce high-performance sports equipment and streamline supply chains.

Virtual and augmented reality (VR/AR) are also prominent Industry 4.0 innovations in sports (14). VR can create immersive training environments (e.g., a quarterback practicing against a virtual defense) or simulators for motorsports and flight. AR overlays, accessed via smart glasses or stadium screens, can provide athletes and fans with enriched real-time information (such as distance sensors for ski jumpers). Moreover, the emerging concept of a digital twin, a virtual replica of a stadium or even a player's physiology, is being explored to model performance and maintenance scenarios (25–27).

However, these developments also bring ethical and social challenges (28). Access to immersive training systems or digital twins is often restricted to wealthy clubs, creating technological divides between elite and grassroots levels. Wearables and camera systems risk becoming tools of surveillance, with continuous monitoring turning athletes into data extraction sites. Such concerns echo broader critiques of “data colonialism,” where human activity is mined and monetized without sufficient agency or consent. A Sports 5.0 perspective must therefore emphasize not only technological innovation but also safeguards for fairness, privacy, and equitable access.

Another fast-growing domain is blockchain and distributed ledger technology: teams are experimenting with blockchain-based fan tokens (like those issued by clubs on Socios) to monetize fan engagement, and major events are investigating blockchain for secure ticketing and anti-counterfeiting.

Glebova et al. (1) emphasize that such technologies are reshaping the sports ecosystem: “from fan engagement to athlete performance, data analytics, and immersive experiences,” (p. 1) innovations like AI and blockchain are “redefining how sport is played, consumed, and commercialized” (p. 1). For example, teams now use AI-powered video analysis tools to tag every action in a match instantly or employ predictive analytics to scout emerging talent. Wearable data feeds are used to monitor players' wellness (e.g., tracking sleep and stress to prevent burnout). On the business side, IoT sensors in stadium infrastructure optimize energy use and crowd flows, while 5G networks enable ultra-high-definition live streaming to remote fans and support VR broadcasts.

However, as these tools proliferate, it is crucial (consistent with a 5.0 ethos) to implement them thoughtfully. Sports scientists and engineers must ensure that AI and big data are transparent and augment human insight, not replace it. Mateus et al. (29) emphasize that AI systems in sports collect large volumes of sensitive information, including biometric, physiological, and personal data—raising critical concerns about data storage, sharing, and usage. They highlight the need for strict compliance with data protection regulations, such as the GDPR, and stress the importance of transparent communication with athletes about how their data will be used. Importantly, they argue that AI tools should support rather than replace human judgment. In practice, this means that coaches and sports scientists incorporate AI-generated insights as one factor among many, ensuring that athletes retain agency and that expert judgment remains central to decision-making.

Table 2 summarizes how Industry 5.0 principles (human-centricity, sustainability, resilience) can be realized through technology in sports. For instance, human-centricity (placing people first) translates to wearable tech designs that prioritize athletes' privacy and comfort, as well as fan engagement platforms that enable co-creation (allowing supporters to have a voice). Sustainability leads to adopting smart stadium systems that minimize waste and energy, and to using analytics to reduce unnecessary travel (e.g., optimizing tournament scheduling). Resilience manifests in digital backbones (30), like cloud-based broadcasting and virtual fans, that keep sports running even when in-person events are disrupted (as seen during COVID-19).

**Table 2:** Industry 5.0 pillars and their application in the sports industry.

Pillar	Industry 5.0 principle	Sports applications	Illustrative cases/stakeholders/outcomes
Human-centricity	Empower humans, fairness, inclusion	Athlete mental health support, accessible tech (AR for disabled athletes), fan co-creation platforms	Example: Simone Biles and Naomi Osaka advocating for mental health leading to institutional reforms; Paralympic AR tools to enhance inclusivity. Outcomes: reduced stigma, broader participation.
Sustainability	Environmental responsibility, circular economy	Green stadium design (solar power, LEED certification), carbon-neutral events, sustainable sports equipment (recycled materials)	Example: Forest Green Rovers (UK) as a carbon-neutral club; F1 commitment to sustainable fuels. Stakeholders: athletes, fans, local communities. Outcomes: reduced emissions, fan awareness.
Resilience	Robustness to crises, adaptability	Virtual attendance and eSports platforms, diversified revenue models, multi-use facilities (sports + community events)	Example: NBA “bubble” tournament during COVID-19 as proof of adaptive design. Grassroots clubs using hybrid digital/physical programs during lockdown. Outcomes: continuity of sport, new revenue, community trust.

Taken together, these examples underline that the promise of Sports 5.0 lies not in replacing human expertise with machines but in using technology as an assistive, ethical, and inclusive tool. The challenge is to ensure that innovations designed for performance and profit do not undermine trust, well-being, or the cultural value of sport.

## 5 Ethics, data, and human agency

With technology deeply embedded, ethical considerations come to the fore (31, 32). Privacy and data rights are paramount: athletes and fans generate rich data, and protecting that information is both a legal and moral obligation. Sports organizations must implement strong data governance. For example, a team using GPS trackers on players must secure consent and clarify how data contribute to performance or health programs. Mateus et al. (29) highlight that respecting data privacy frameworks (like GDPR) and maintaining transparency are critical to maintaining trust and ensuring ethical practices.

Yet the proliferation of athlete data also raises deeper concerns that extend beyond compliance. Scholars in the sociology of sport argue that continuous biometric monitoring represents a new form of surveillance, where bodies are quantified, compared, and managed according to corporate or institutional logics (33, 34). For example, professional footballers have voiced discomfort about GPS trackers revealing their physical condition to coaches, scouts, and sponsors, potentially affecting career opportunities. Rather than empowering athletes, such practices risk reinforcing asymmetries of control, with data primarily serving organizational or commercial interests.

Equally important is ensuring that technology amplifies, rather than undermines, human values. There is a growing debate about the ethics of AI decision-making in sports—from automated officiating to lineup choices. Algorithms can inherit biases (just as any human might), so sports technologists must guard against unfairness. For instance, if AI scouting tools undervalue athletes from certain regions or backgrounds due to biased training data, this would perpetuate inequity. Adopting human-centric design can mitigate this: AI systems in sport should be regularly audited, with human experts overseeing critical calls. In line with Industry 5.0's ethos, we argue (as others have) that AI should support coaches' intuition and athletes' development rather than replace the human touch (29). A practical illustration is the use of AI scouting platforms that rank youth players. While marketed as objective, these systems often rely on training data skewed toward certain regions or playing styles, disadvantaging athletes from underrepresented contexts. In this sense, algorithmic tools can reproduce old inequalities under the guise of innovation. Embedding regular audits and athlete participation in system design is crucial if Sports 5.0 is to live up to its human-centric ethos.

Ethical sports management also extends to labor and equity. The European Commission's vision of Industry 5.0 stresses worker well-being (2, 3); similarly, sports organizations must ensure that behind-the-scenes staff (trainers, facility workers, stadium staff) have decent conditions, and are empowered by technology through upskilling programs. Automation should not simply displace workers but create new roles (e.g., data analysts in coaching teams). Moreover, modern sports operations—like all industries—must incorporate inclusive design: venues should be accessible to people with disabilities, and technology should not exclude non-tech-savvy fans (e.g., by ensuring digital ticketing has non-digital alternatives).

Ethical responsibility also extends to recognizing the boundaries of technological legitimacy in sport. Not all aspects of play can or should be optimized through data. Elements of uncertainty, spontaneity, and human error are integral to the meaning of sport as a cultural practice (35, 36). Over-reliance on technological mediation risks hollowing out these qualities. Sports 5.0, therefore, must embrace a dual responsibility: to protect athletes and fans from exploitation, and to preserve the cultural and experiential integrity of sport itself.

## 6 Fan culture and digital engagement

The rise of digital media has transformed fan culture (37, 38). Fans today are not passive spectators, but active participants connected through global networks. Romero-Jara, Solanellas, Muñoz, and López-Carril [(39), p. 2] observe that in football, digital technologies have shifted from being optional extras to becoming central components of clubs' strategy and operations, as fans now expect continuous engagement with their teams through platforms like social media, podcasts, and eSports. In a Sports 5.0 world, meeting these fan expectations must be balanced with responsible practices.

One example is the rise of blockchain-based fan tokens on platforms such as Socios, where clubs like Paris Saint-Germain and FC Barcelona give supporters limited voting rights in club decisions. While marketed as democratizing engagement, these tokens often privilege fans with financial means, turning participation into an investment product. In Japan, baseball teams have experimented with interactive live-streaming platforms that allow remote fans to influence in-game entertainment, demonstrating how technology can reshape fan rituals. Meanwhile, in African football, mobile-based fan engagement apps provide access to scores, merchandise, and community content in regions where stadium attendance may be limited. These examples illustrate that fan “co-creation” is mediated by social, economic, and technological conditions rather than being universally accessible.

Social platforms (Twitter, Instagram, TikTok, etc.) allow teams to engage fans across ages and geographies (40). Clubs invest in social media content to build their brand and community. For example, a club might stream behind-the-scenes videos, host interactive Q&As, or even let fans vote on minor decisions. In turn, analytics gleaned from these interactions help personalize experiences. However, Sports 5.0 cautions against manipulation: data-driven marketing should respect fan privacy and avoid exploitative profiling. Fans should also have a voice: some franchises have launched fan tokens (blockchain-based digital assets) that give supporters a say in club matters. This co-creation of content and community is a hallmark of a human-centered approach.

Online communities break down geographical barriers. A Liverpool fan in Tokyo or an Al Ahly supporter in Nairobi can form communities online. Technology like augmented reality (e.g., AR filters, virtual stadium tours) can enhance these communities' feeling of participation. At the same time, leagues must guard against toxicity and online harassment, which digital anonymity can amplify. Sports bodies should proactively moderate social channels to ensure fan spaces are inclusive and respectful. At the same time, global inequalities persist. Digital engagement assumes access to stable internet, modern devices, and disposable income for subscriptions or tokens—conditions that exclude large segments of the global fan base. Online toxicity, including racist abuse of athletes on platforms such as Twitter and Instagram, highlights how digital spaces can reproduce social harms rather than alleviate them. Sports 5.0 requires not only technical safeguards but also governance measures that ensure fan cultures are safe, inclusive, and genuinely participatory.

Fan engagement in a Sports 5.0 framework must therefore be understood as a global and uneven process. Whereas elite clubs in Europe experiment with NFTs and VR stadium tours, grassroots organizations in Latin America or Africa rely on low-bandwidth mobile platforms to connect with their communities. Recognizing these asymmetries prevents Sports 5.0 from being portrayed as a universal experience when, in practice, it is shaped by local resources, cultures, and governance structures.

## 7 Inclusion, diversity, and social justice

A major pillar of Sports Industry 5.0 is ensuring that sports serve all people fairly and respectfully (41, 42). In recent years, athletes have increasingly used their platforms to address systemic issues. As one analysis notes, “From Colin Kaepernick taking a knee to Simone Biles speaking candidly on the importance of prioritizing mental health, athletes continue to use their power and platforms as cultural influencers to […] tip the scales towards justice and equity” (43). In a 5.0 framework, organizations should welcome this engagement, rather than stifle it, because athletes' advocacy aligns sport with broader social progress.

These dynamics are not confined to Euro-American contexts. In New Zealand, the integration of Māori traditions into rugby underscores how indigenous values can reshape sporting culture. In South Africa, rugby and football clubs have used inclusion campaigns to confront the legacy of apartheid, linking sport with broader reconciliation agendas. In India and Brazil, community-based programs provide access to girls and young people in low-income areas, often using sport as a pathway to education and empowerment. Such examples show that inclusion must be interpreted locally if Sports 5.0 is to be globally relevant.

Diversity and equity must permeate every level of sports. This includes gender equity (equal pay and investment in women's sports, equitable facilities), racial equity, support for LGBTQ + athletes, and disability sports. For example, governing bodies are wrestling with inclusion of transgender athletes: policy frameworks (like the IOC's Fairness, Inclusion and Non-Discrimination policy) emphasize human-rights based approaches. Similarly, anti-racism initiatives are critical. High-profile incidents of discrimination have spurred calls for deeper action: national federations and clubs are making formal commitments to acknowledge underrepresented groups (such as people of color, indigenous communities and women) in leadership and participation.

However, inclusion efforts cannot be divorced from the structural problems that continue to undermine sport. Abuse scandals in youth gymnastics, corruption in FIFA, and financial inequities between men's and women's leagues highlight how deeply entrenched power imbalances remain. Without addressing these systemic failures, initiatives framed as “diversity” or “equity” risk becoming symbolic gestures rather than substantive reforms. Sports 5.0 must therefore be understood not only as a technological and cultural shift but also as a governance challenge requiring transparency, accountability, and independent oversight.

Good governance underlies these efforts. Industry 5.0's human-centric governance means transforming sports organizations to be truly accountable and representative. The Centre for Sport and Human Rights (44) emphasizes that leaders must fully integrate human rights in sport governance and culture, building diversity in management and independent oversight. This can involve creating social “licenses” by engaging athletes, fans, and communities in decision-making, and ensuring transparent remedies when harm occurs. The misconduct scandals of recent years (from abuse in youth sports to corruption in international bodies) highlight the need for proactive culture change: sports must foster humility, transparency, and empathy in management.

Concretely, inclusion also means expanding access. Community sports programs should address social barriers: outreach in underserved neighborhoods, subsidized youth leagues, gender-neutral facilities, and adaptive sports for people with disabilities. Technology can aid this: mobile training apps, online coaching clinics and remote community hubs can bring sports to people who lack traditional infrastructure. But all such initiatives must be culturally sensitive, respecting local values and languages while promoting universal sporting values. In sum, Sports Industry 5.0 views social justice not as an optional add-on but as integral, echoing the idea that sports, given their global reach, are a “low-cost, high-impact tool” for social change.

In practical terms, this means embedding human rights standards into the everyday governance of sport, ensuring that leadership structures reflect the diversity of athletes and fans they represent, and creating accessible mechanisms for redress when harm occurs. Only by linking inclusion to governance reform can Sports 5.0 avoid being seen as a mere rhetorical upgrade and become a meaningful pathway toward justice and equity.

## 8 Athlete mental health and well-being

The spotlight on mental health in sports has grown dramatically. Elite athletes face intense pressure, and stigma often prevents seeking help. Sports Industry 5.0 implies a duty of care: athletes are human beings first, and their psychological well-being matters at least as much as physical fitness. The importance of this shift is underscored by high-profile cases. Simone Biles' decision to withdraw from Olympic events in 2021 and Naomi Osaka's withdrawal from the French Open in the same year both brought unprecedented visibility to the mental strain faced by elite athletes. These moments challenged traditional norms that equated mental resilience with silence and highlighted the need for structural changes in how sports organizations support psychological well-being. Their choices generated public debate, signaling that athlete welfare is not only a personal issue but also an institutional responsibility. Technology can support this shift. The pandemic underscored the mental toll of isolation on athletes and youth, creating an impetus for new solutions. Balcombe and De Leo (45) argue that digital mental health platforms and algorithms (machine learning, AI) can inform prevention and early intervention strategies and are a “critical success factor in a post-COVID-19 world” (p. 11). For example, apps using AI chatbots or virtual counseling can provide confidential support to athletes. Wearables and mobile sensors could passively monitor signs of stress or depression, flagging coaches or psychologists when intervention is needed. Such systems must be designed with care (avoiding false alarms or privacy intrusions), but they offer promise for timely help.

While digital platforms and wearables hold promise, they also introduce risks. Overreliance on algorithmic monitoring could reduce athletes' autonomy, while constant surveillance may exacerbate anxiety rather than relieve it. Confidentiality is another challenge: sensitive biometric or psychological data may be misused in contract negotiations or sponsorship decisions. These concerns suggest that digital tools should be integrated only as part of broader human-centered systems, with strong safeguards and clear boundaries around consent and data ownership.

Beyond tech, Sports 5.0 stresses integrated support structures. This means funding sport psychologists, embedding mental health education in training programs, and normalizing conversations about stress and burnout. Athletes with visible struggles have become spokespeople that shift norms: the American Psychological Association notes that around 35% of elite athletes report mental health issues, comparable to the general population; the difference is that sports have unique stressors (45). Removing stigma and building resilience are thus core to future sports management. The sports industry should leverage digital tools as complements (for screening, anonymous self-help, teletherapy) but ultimately foster sports cultures where asking for help is seen as strength, not weakness.

Research indicates that mental health symptoms of anxiety, depression, or related conditions are common in elite athletes (46–48). While figures are relatively similar in the general population, the specific pressures of competitive sport—intense public scrutiny, precarious contracts, and relentless schedules—make athletes particularly vulnerable. Addressing these realities requires more than digital innovation: it calls for embedding mental health professionals within teams, normalizing open conversations about stress, and ensuring that institutional policies protect rather than penalize those who seek help (49, 50). Within Sports 5.0, athlete well-being should be treated not as an optional benefit but as a fundamental component of organizational legitimacy.

## 9 Sustainability and environmental responsibility

Climate change and ecological crises confront every industry—and sports is no exception (51, 52). Paradoxically, while sport can inspire environmental awareness, it also has a substantial footprint. The United Nations Department of Economic and Social Affairs reports that the “global sport sector contributes the same level of emissions as a medium-sized country,” (p. 1) with major events like the Olympic Games and FIFA World Cup emitting millions of tons of CO₂ (e.g., Rio 2016: ∼3.6 million tons, Russia 2018: ∼2.2 million tons) (53). Moreover, extreme weather events are already disrupting sports schedules (e.g., heat breaks in tennis, cancelled ski events due to lack of snow), indicating that sports is both contributor to and victim of climate change.

Recent events illustrate both progress and tension. The Tokyo 2020 Olympics used medals made from recycled electronics and deployed hydrogen-powered vehicles, yet still generated significant emissions from international travel and construction. Forest Green Rovers in the UK have branded themselves as the world's first carbon-neutral football club, adopting vegan menus and solar-powered facilities as part of a comprehensive environmental strategy (54). Formula 1 has announced plans for sustainable fuels and net-zero operations by 2030 (55). These initiatives show how sustainability measures can be implemented at multiple levels but also expose the difficulty of reconciling mega-events with climate goals.

Sports Industry 5.0 mandates that the sector confront these realities proactively. This involves greening the infrastructure: building energy-efficient, zero-carbon stadiums; sourcing renewable energy; implementing water recycling and waste reduction at venues. Many organizations are moving in this direction. For instance, the International Olympic Committee adopted environment as a pillar of Olympism in 1994 and now requires host cities to offset carbon and preserve biodiversity. Some leagues and teams have unveiled climate commitments (European football clubs aiming for carbon neutrality; F1 developing sustainable fuels).

Beyond doing less harm, sports can be a powerful platform for sustainability education. The UN notes that sports' broad appeal makes it a low-cost, high-impact tool (p. 2) for raising awareness of global warming (53). Athletes and teams increasingly act as climate ambassadors. At COP26 in 2021, over 50 Olympic and Paralympic athletes urged leaders for action, leveraging their influence with fans. Events can embed green messages (recycling campaigns at matches, half-time climate awareness presentations). Research even suggests that fans attending events with visible eco-initiatives are more willing to adopt sustainable habits themselves. In a Sports 5.0 vision, every match or race becomes a micro-cosmos for environmental leadership.

However, scholars of sport and sustainability caution against conflating high-profile gestures with structural change. Purchasing carbon offsets while maintaining intensive travel schedules or using “green” branding without systemic reform risks constituting greenwashing. Without independent audits and transparent reporting, even ambitious sustainability claims may mask continued environmental harm. Sports 5.0 must therefore encourage robust metrics, third-party verification, and stakeholder engagement to ensure that environmental commitments translate into real impact.

Beyond elite sport, sustainability also means supporting grassroots infrastructure that is climate-resilient and low-carbon. In low-resource settings, this can involve community-built facilities using local materials, shared spaces for multiple activities, and digital coordination to reduce travel. Including these contexts prevents Sports 5.0 from remaining a top-down project and anchors it instead in diverse practices worldwide.

## 10 Strategic innovation and governance

To navigate the 5.0 transition, sports organizations must adopt new business models and governance structures (56, 57). Digitization opens revenue streams: direct-to-consumer streaming services, eSports spin-offs, gamified fan experiences, and data-driven personalization. For example, leagues now offer subscription video-on-demand packages (NBA League Pass, Premier League streaming) leveraging global fan bases. Partnerships with tech firms and broadcasters extend reach; Deloitte notes that integrating technology can open up new revenue streams as sports brands become content creators across multiple platforms (58). NFTs, virtual merchandise, and fantasy sports further diversify income.

Yet commercial innovation is not evenly distributed. While major leagues can invest in direct-to-consumer streaming or NFTs, many women's leagues and smaller federations struggle to secure even basic broadcasting rights. Financial inequities remain stark: for example, despite rising viewership of women's football, sponsorship and media revenues still lag far behind men's competitions. Without deliberate redistribution, digital transformation may amplify these gaps rather than close them.

Yet these commercial shifts must align with strategic values. Sports 5.0 implies responsibly managing digital growth. Leagues must wrestle with issues like gambling ads, celebrity-only endorsements, and the exclusivity of technology (not pricing out traditional fans). Flexibility is key: the COVID era showed how quickly sports can be disrupted (59, 60), so organizations are exploring hybrid event models (combining limited in-person attendance with extensive virtual experiences) to stay resilient.

Governance-wise, major sports bodies face intense scrutiny. We have argued above that human rights due diligence and ethical leadership are non-negotiable. This will shape strategic directions. For instance, boycotts over human rights (as seen around certain mega-events) pressure governing bodies to set standards on workers' rights, freedom of expression, and social equity. Sports 5.0 leaders will need to be proactive, not just reactive: embedding social and environmental criteria in decision-making (e.g., choosing host cities with sustainable proposals, vetting sponsors for ESG compliance). Organizations may adopt sustainability reporting (ESG metrics) as standard practice.

Governance challenges are not theoretical. Corruption scandals in FIFA, abuse cases in gymnastics, and ongoing concerns about labor rights in mega-event construction illustrate the entrenched problems that undercut credibility. While Sports 5.0 emphasizes human-centric and ethical governance, these principles must be backed by independent oversight and enforcement. Otherwise, commitments risk becoming aspirational rhetoric that leaves structural abuses unaddressed.

At the national and community level, governments and NGOs will influence the transition. Policies supporting community sport infrastructure, inclusive programs, and athlete education are part of the ecosystem. For example, climate policies might offer incentives for green stadiums; labor laws may require better athlete protections. Multi-stakeholder collaborations (e.g., UNESCO's Sports for Development initiatives, or the “Green Sports Alliance” industry coalition) exemplify the direction of the field. Ultimately, strategic innovation in Sports Industry 5.0 means aligning competitive success with ethical, environmental and social goals, a proposition that may sound countercultural, but which the crises of our time demand.

In practical terms, this means adopting transparent ESG reporting, ensuring independent auditing of human rights commitments, and creating formal avenues for athlete and fan representation in governance. Multi-stakeholder coalitions such as the Green Sports Alliance or the Centre for Sport and Human Rights demonstrate emerging models. Sports 5.0 governance must therefore be judged not only by innovation or vision but by its ability to deliver accountability in contexts historically resistant to reform.

## 11 Critical reflections: limits, tensions, and cautions of sports 5.0

While earlier sections highlighted challenges related to technology, governance, and sustainability, it is useful to synthesize these into a broader reflection on the structural limits of Sports 5.0.

The Sports Industry 5.0 framework presents a compelling vision of ethical, inclusive, and sustainable transformation, but it is not without limitations and potential contradictions. Critics may argue that the concept risks falling into the trap of techno-utopianism (61, 62), where the promise of innovation overshadows the structural inequalities and power asymmetries that remain deeply embedded in global sport.

Despite its human-centered rhetoric, much of the technology underpinning Sports 5.0—AI, wearables, blockchain, immersive media—is expensive, data-intensive, and controlled by powerful private interests. This raises concerns about access and equity. Elite clubs and wealthy leagues can afford to adopt cutting-edge systems, but grassroots organizations in the Global South or low-income communities often face exclusion or are left behind. A 5.0 vision without redistribution risks entrenching a two-tier sports system, where innovation serves a privileged few while others remain digitally marginalized.

Another concern is the rise of performative sustainability (63). Many sports entities now market carbon offsets, green venues, or ESG goals, but critics question whether these actions are more symbolic than systemic (64). Buying carbon credits while flying teams around the globe, or installing solar panels at stadiums without revisiting consumption habits, can amount to little more than greenwashing. Similarly, commitments to diversity or athlete mental health may remain superficial if they are not supported by structural reforms, adequate funding, and effective accountability mechanisms.

The datafication and quantification of sport (33, 65) also raises risks of surveillance and control (66). As athletes become sites of continuous biometric monitoring, and fans generate behavioral data used for microtargeted marketing, the line between empowerment and exploitation blurs. Some scholars have described this trend as data colonialism—the extraction of human data for profit, often without meaningful consent or oversight (67). In this light, the Sports 5.0 emphasis on “human-centric” technology may mask how human lives are increasingly engineered, commodified, or managed through opaque systems.

While Sports 5.0 emphasizes ethical leadership and participatory governance, critics may point out the persistent lack of regulatory enforcement in the sports world. From FIFA to the IOC to national federations, many institutions have struggled with transparency, labor rights, and abuse scandals (68, 69). Without external accountability or independent oversight, the values of Sports 5.0 may remain more aspirational than operational. Additionally, emerging technologies often outpace existing governance structures, leaving legal and ethical vacuums that can be exploited.

Finally, the 5.0 framework could unintentionally reinforce a hyper-managerial logic (70, 71), where every aspect of sport—performance, recovery, fan sentiment, even community engagement—is measured, optimized, and made legible to analytics. This risks reducing sport to a system of KPIs and dashboards, undermining its cultural, emotional, and spontaneous qualities. As sports become platforms for data accumulation and predictive modeling, there is a danger of losing the *play* in play.

In sum, while Sports Industry 5.0 offers a compelling roadmap for transformation, it should not be adopted uncritically. Without addressing the deep-rooted power dynamics, regulatory gaps, and socio-economic disparities that define the sports world, it may become yet another rhetorical upgrade, promising inclusion, ethics, and sustainability while delivering more of the same, in sleeker packaging. There is also the danger that 'Sports 5.0' becomes a managerial slogan or buzzword (70, 72) rather than an analytical concept, adopted by organizations to signal progressiveness without altering core practices. Therefore, the critical test is whether Sports 5.0 can transcend aspirational language and address entrenched structural inequities. Without this, it risks becoming another iteration of managerial rhetoric rather than a transformative paradigm (cf. [e.g. (6)].

## 12 Conclusion

The idea of “Sports Industry 5.0” compels us to reimagine the future of sports. It is not a fixed blueprint, but a guiding ethos: that the next era of sports must weave together advanced technology and human values. This paper has argued that embracing Sports 5.0 principles—human-centric design, sustainability, resilience, and justice—will be essential for the sports sector's legitimacy and survival. We have surveyed how Industry 4.0 tools (AI, IoT, VR, data analytics, etc.) can be harnessed to elevate performance, safety and fan engagement, while also highlighting the need to guard against ethical pitfalls. We have emphasized that sports are a microcosm of society; thus, innovations in the field should promote mental health, diversity and environmental stewardship just as vigorously as they chase wins and profits.

In practical terms, Sports Industry 5.0 will see training facilities powered by smart, green technologies, broadcasting that connects global audiences ethically, and stadiums operating as net-zero energy systems. Athletes will receive data-driven coaching that respects their autonomy and privacy; fans will participate through digital platforms that value their feedback and wellbeing. Grassroots sports will leverage mobile technology to expand access, and leagues will govern with transparency and equity. These transformations will not happen automatically; they will require bold leadership, cross-sector collaboration, and a willingness to prioritize long-term societal impact over short-term gains.

Ultimately, the sports sector must learn from recent crises: a pandemic, social upheavals, and climate disasters have already forced unprecedented adaptations (bubble tournaments, athlete protests, hastily built sustainable venues). We propose that embracing Sports Industry 5.0 is the proactive path forward—turning today's challenges into an opportunity for renewal. As the UN observed, sport's “potential as a paradigm of sustainable development” has been recognized globally. The question now is whether sports leaders will seize this moment to retool the industry with conscience and creativity.

Future research should test and refine the Sports 5.0 framework through comparative and empirical studies. Cross-cultural case analyses could examine how principles of human-centricity and sustainability are interpreted in different contexts, from elite leagues in Europe to grassroots programs in Africa, Asia, and Latin America. Longitudinal studies could trace whether digital innovations in sport deliver lasting improvements in equity and well-being, or whether they reproduce existing hierarchies. Empirical work on data governance, athlete surveillance, and fan engagement is especially needed to evaluate whether “human-centric” rhetoric is matched by practice. Finally, governance research should investigate how transparency, anti-corruption reforms, and athlete representation can be institutionalized. Such inquiries will help determine whether Sports 5.0 is a transformative paradigm or a normative vision struggling against entrenched structures. If pursued critically and inclusively, Sports Industry 5.0 can provide a pathway for sport to remain a source of inspiration, unity, and joy—while also addressing the urgent ethical, environmental, and social demands of the 21st century.
